# Synthesis and Properties of Monolayer MnSe with Unusual Atomic Structure and Antiferromagnetic Ordering

**DOI:** 10.1021/acsnano.1c05532

**Published:** 2021-07-27

**Authors:** Markus Aapro, Md. Nurul Huda, Jeyakumar Karthikeyan, Shawulienu Kezilebieke, Somesh C. Ganguli, Héctor González Herrero, Xin Huang, Peter Liljeroth, Hannu-Pekka Komsa

**Affiliations:** † Department of Applied Physics, Aalto University, 00076 Aalto, Finland; ‡ Rajiv Gandhi Institute of Petroleum Technology, Jais, Amethi 229304, Uttar Pradesh, India; § Microelectronics Research Unit, University of Oulu, 90014 Oulu, Finland

**Keywords:** 2D materials, magnetism, transition metal chalcogenides, scanning tunneling microscopy, density functional theory

## Abstract

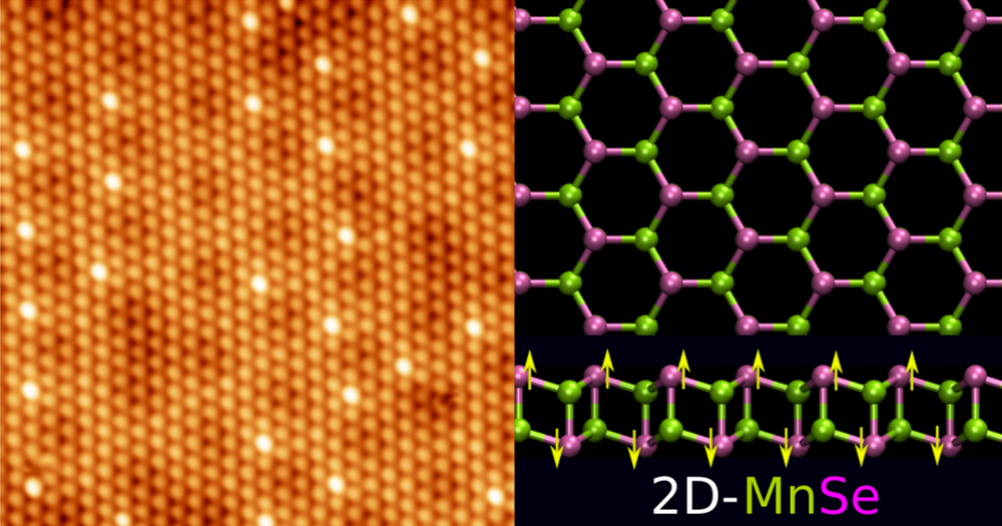

Transition metal chalcogenides (TMCs) are a large family of 2D materials that are currently attracting intense interest. TMCs with 3d transition metals provide opportunities for introducing magnetism and strong correlations into the material with manganese standing out as a particularly attractive option due to its large magnetic moment. Here we report on the successful synthesis of monolayer manganese selenide on a NbSe_2_ substrate. Using scanning tunneling microscopy and spectroscopy experiments and global structure prediction calculations at the density functional theory level, we identify the atomic structure and magnetic and electronic properties of the layered Mn_2_Se_2_ phase. The structure is similar to the layered bulk phase of CuI or a buckled bilayer of *h*-BN. Interestingly, our results suggest that the monolayer is antiferromagnetic, but with an unusual out-of-plane ordering that results in two ferromagnetic planes.

## Introduction

Transition metal chalcogenides (TMCs) are an important and large family of 2D materials with the most famous members belonging to the transition metal dichalcogenides (*e.g.*, the prototypical MoS_2_). In addition to dichalcogenides, also monochalcogenides (such as FeSe and GaSe) and trichalcogenides (such as TiS_3_) have been widely studied. These materials host a broad variety of different kinds of physical phenomena such as valley physics, strong excitonic effects, superconductivity, charge-density wave (CDW) states, and topological phases of matter.^1−4^

Compounds of 3d transition metals are particularly interesting due to the possibilities of introducing magnetism and strong correlations. Several layered 3d-TMCs have been experimentally synthesized, such as TiX_2_ (X = S, Se, or Te), TiX_3_, VX_2_, CrTe_2_, MnSe_2_, FeSe, FeTe, FeTe_2_, CoTe_2_, and NiTe_2_.^5−13^ Out of these, TiX_2_, CoTe_2_, FeTe_2_, and NiTe_2_ are nonmagnetic and FeSe is antiferromagnetic. Ferromagnetic ground states have been computationally predicted for VSe_2_, CrTe_2_, and MnSe_2_,^14,15^ but experimentally the situation is still far from clear. In the case of CrTe_2_, it is difficult to grow high-quality monolayers due to Cr self-intercalation.^13^ As for VSe_2_, there are several reports indicating either the presence or the lack of ferromagnetism.^16−19^

Mn chalcogenides, on the other hand, have received much less attention. Mn can have a maximum magnetic moment of 5 μ_B_ in its elemental form, and thus it presents an attractive route toward 2D magnetic materials by Mn doping of nonmagnetic 2D materials.^20,21^ Such materials would be promising for magnetism and spintronics applications and have also been targeted for the realization of exotic quantum phases such as quantum anomalous Hall insulators.^22−25^ However, reports of 2D phases of manganese chalcogenides appear to be rare. Most commonly reported manganese chalcogenides are thin films of the α-phase MnS/MnSe, which has a rock-salt structure. In these studies, the film surface is cleaved normal to the (111) direction and from the side view the structure resembles the T-phase of dichalcogenides. Computational studies suggested that MnS_2_ and MnSe_2_ should be stable in the T-phase and are predicted to be strongly ferromagnetic, with a high *T*_C_ of about 250 K and μ = 3 μ_B_.^14,26^ Experimentally, Li *et al.* reported thin films of α-MnS down to thickness of 4.78 nm.^27^ O’Hara *et al.* reported synthesis of both thin films of α-MnSe and likely also a monolayer of T-MnSe_2_.^8^ The monolayer samples showed signs of ferromagnetism in agreement with the computational predictions.

Here we report on the successful synthesis of monolayer manganese selenide on NbSe_2_ substrate. Using scanning tunneling microscopy (STM) and spectroscopy (STS) experiments and global structure prediction calculations at the density functional theory (DFT) level, we identify the material to possess an unusual atomic structure similar to that found in the layered bulk phase of CuI. We also investigate the electronic and magnetic properties of the material, as well as its point defects. Interestingly, the calculations (supported by experiments) show that the monolayers are antiferromagnetic but with out-of-plane ordering that results in two ferromagnetic planes. The spin degeneracy can be lifted upon application of an electric field, and we thus envision this material to be promising for applications in spintronics, *e.g.*, in spin filtering.

## Results and Discussion

The MnSe monolayer was grown on bulk NbSe_2_ substrate by e-beam evaporation of Mn and co-deposition of Se from a Knudsen cell in ultrahigh-vacuum (UHV) conditions. Before growth of the MnSe, the bulk NbSe_2_ was cleaved inside the vacuum chamber and degassed. During the growth of the MnSe layer, the substrate was kept at 490 K temperature. The Mn flux and Se flux were directed to the surface of the NbSe_2_ substrate. The growth of the MnSe is determined by the Mn flux, and the excess selenium desorbs from the substrate since the substrate temperature was much higher than the evaporation temperature of selenium atoms (*T* = 393 K) in UHV conditions. After deposition, the sample is annealed in a Se-rich environment.

**Figure 1:**
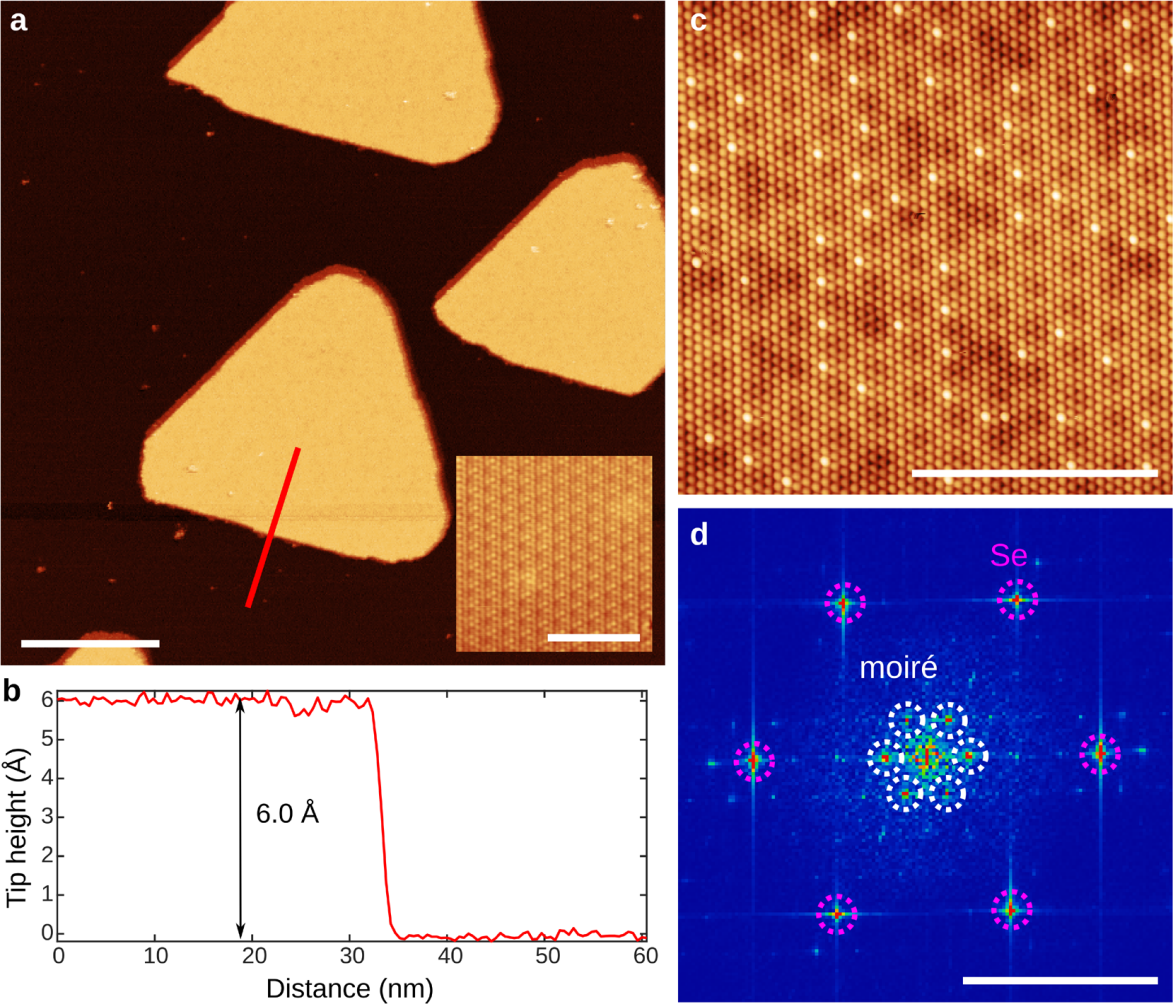
(a) Large-area STM scan showing the MnSe islands on NbSe_2_ substrate. *V*_bias_ = −1 V, *I* = 5 pA, and scale bar = 50 nm. Inset: atomic resolution scan of the NbSe_2_ substrate. *V*_bias_ = −78.5 mV, *I* = 500 pA, scale bar = 5 nm. (b) Line profile along the red line shown in panel a. (c, d) Atomic resolution scan (c) and the corresponding 2D-FFT (d) on a MnSe island. *V*_bias_ = −157.5 mV, *I* = 500 pA, and scale bar = 5 nm. The peaks corresponding to the moiré pattern and atomic lattice are highlighted with white and purple, respectively. Scale bar = 3 nm^–1^.

Figure 1a shows a large-scale STM image of monolayer MnSe islands with atomically sharp edges grown on NbSe_2_ substrate. The atomic resolution topography shows that the NbSe_2_ surface exhibits a typical CDW pattern.^28^ The line profile taken over the islands (see Figure 1b) depicts that the apparent thickness of the layer is 6.0 Å, a typical value for monolayers of transition metal (di)chalcogenides.

Atomically resolved STM images of the MnSe surface, as shown in the Figure 1c, reveal a triangular atomic lattice with a lattice constant of 4.3 Å. This already gives a strong indication that the MnSe island is not of T-phase MnSe_2_, since its lattice constant of 3.5 Å is markedly different. The lattice mismatch and the relative angle between NbSe_2_ and MnSe lattices result in the formation of a moiré pattern, which is visible as a large-wavelength background variation in the STM topography. The wavelength can be analyzed by 2D-FFT as shown in Figure 1d (see Supporting Information (SI) Figure S1 for details). The periodicity of the moiré pattern is 18.1 Å with a 0.8° relative angle between NbSe_2_ and MnSe lattices, which are consistent with the observed lattice constants of NbSe_2_ and MnSe. Point defects with high contrast are also visible on all islands: the defect sites largely coincide with high-intensity points of the moiré pattern and they are located on the top sites with respect to the underlying atomic lattice.

To demonstrate the reproducibility of our growth process, we successfully synthesized more samples in another vacuum system and studied them with STM and XPS, SI Figures S4 and S6. The samples exhibited the same properties as the previous ones in terms of lattice constants, high-contrast impurities, and electronic properties as probed by STS. Growth on HOPG substrate, however, was unsuccessful, possibly due to insufficiently low growth temperatures; *cf.*Table S2.

**Figure 2:**
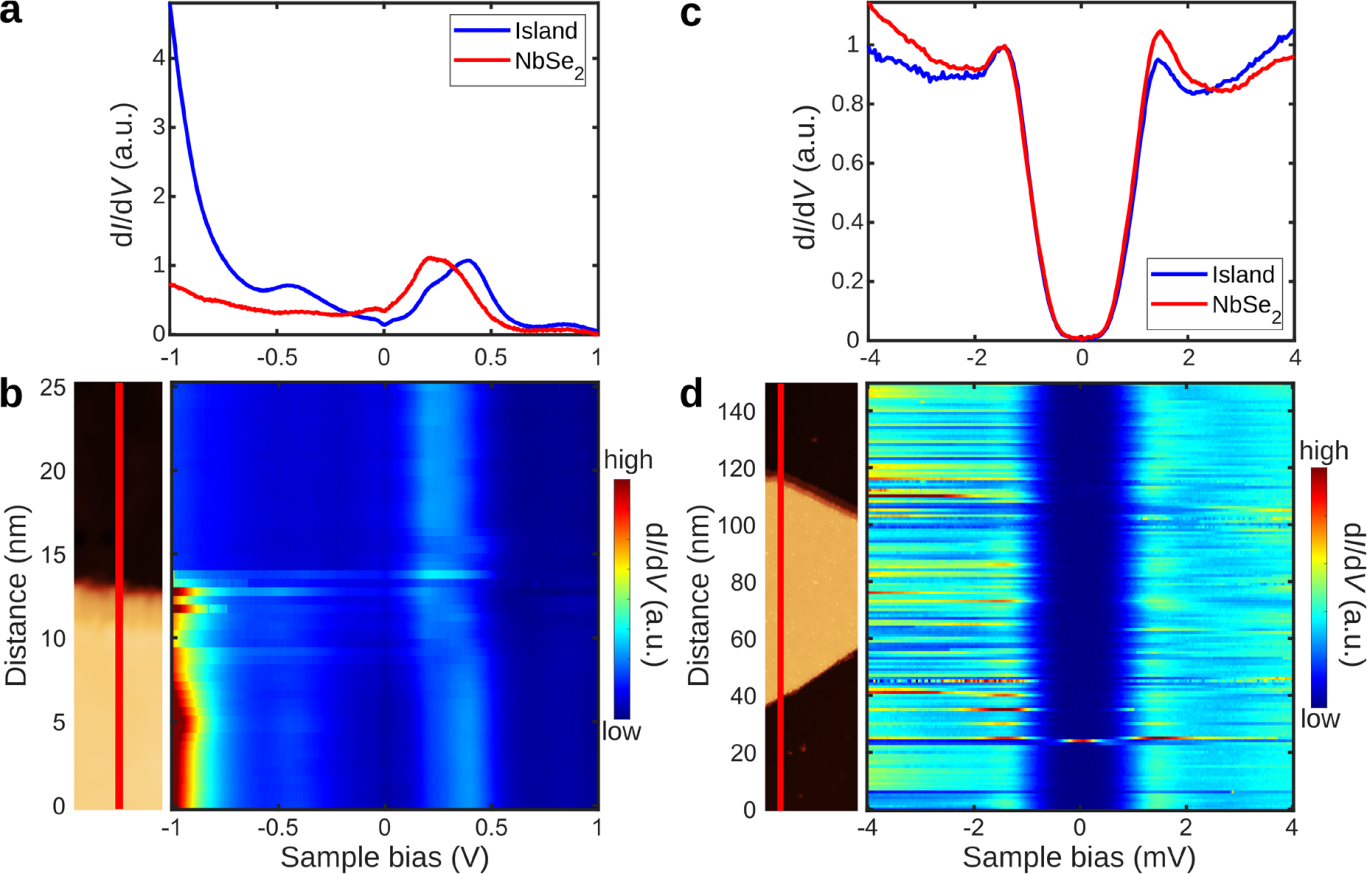
STS of MnSe/NbSe_2_ heterostructures: (a) Averaged d*I*/d*V* spectra on a wide energy range. Voltage modulation 10 mV. (b) Line spectra corresponding to panel a, averaged over 5 lines from grid spectra. Lines were shifted spatially to account for the angle between the island edge and line direction. *V*_bias_ = 0.3 V and *I* = 80 pA for the scan. (c) Averaged d*I*/d*V* spectra of the superconducting gap on an island and NbSe_2_ substrate. (d) Line spectra corresponding to panel c taken along the red line in the inset. *V*_bias_ = 1 V and *I* = 20 pA for the scan.

To investigate the electronic properties of MnSe islands, we conducted STS measurements on MnSe/NbSe_2_ heterostructures. The overall electronic structure of the heterostructure can be assessed by comparing averaged d*I*/d*V* point spectra (see Figure 2a) taken on the MnSe islands (blue curve) and NbSe_2_ substrate (red curve) over a large bias range. We will first focus on the electronic response of the NbSe_2_ substrate. At the positive bias region, the most pronounced features are the broad resonances at around 0.3 V, which mostly arise from Nb-derived d-band, while at the negative bias, the d*I*/d*V* signal is broad and slowly rising.^19,28^ On the other hand, the d*I*/d*V* spectra taken on MnSe reveals that two prominent features arise at bias ∼ ±0.4 V. The electronic features at −0.4 V are likely to emerge from MnSe-related states (see below for a detailed discussion and a comparison with DFT calculations). In the positive bias regime, the features arise from the Nb-derived d-band of the NbSe_2_ substrate that is shifted slightly to higher energies in the presence of the MnSe island. Apart from these low-bias features, the high intensity starting from ∼−0.7 V results from the valence band of the MnSe island. Large bias range spectra taken over a line across the edge of a MnSe island, as shown in Figure 2b, also show that the electronic feature at *ca.* −0.4 V on MnSe island disappears in the bulk NbSe_2_ substrate, while, in the positive bias regime, the energy shift of the Nb-derived d-band is clearly visible. The absence of MnSe-related features at positive bias suggests that the MnSe valence band maximum (VBM) is close to 0 V, with the band gap extending above 1 V. An extended-range d*I*/d*V* spectrum in Figure S5b shows states at 1.8–2.0 V on the islands, which could indicate the onset of the MnSe conduction band.

The potential magnetic properties of the MnSe layer can be assessed by carrying out detailed d*I*/d*V* spectroscopy experiments focusing on the superconducting gap of the NbSe_2_ substrate, as shown in Figure 2c. The gap of NbSe_2_ under the MnSe island is essentially identical to the NbSe_2_ substrate in contrast to earlier experiments with VSe_2_/NbSe_2_ and CrBr_3_/NbSe_2_ heterostructures.^19,29,30^ In addition, we do not observe in-gap Yu–Shiba–Rusinov bands associated with magnetic structures on superconductors. High-resolution line spectra acquired over a MnSe island, as illustrated in Figure 2d, show that there are no discernible spatial variations in the low-bias spectra on the MnSe islands or their edges. No edge states emerge inside the superconducting gap, and the local variations of the gap width are negligible. We have also measured d*I*/d*V* spectroscopy under an external out-of-plane magnetic field. We observe Abrikosov vortices in the d*I*/d*V* maps, and the periodicity of the vortex lattice does not change over the MnSe islands (see Figure S2). The absence of in-gap states and negligible modulation of the superconducting gap imply that the MnSe islands are not ferromagnetic.^19,31,32^

In order to identify the composition and the atomic structure of the synthesized MnSe layer, we employ automated structure prediction with first-principles calculations. We have used Universal Structure Predictor: Evolutionary Xtallography (USPEX) code^33^ to find the lowest energy 2D structure, for a fixed number of Mn and Se atoms and maximum layer thickness set to 7 Å. Since the compositions of Mn and Se in experimentally observed 2D Mn–Se layers remain undetermined, we repeat the calculations for several compositions: Mn_5_Se_2_, Mn_4_Se_2_, Mn_5_Se_3_, Mn_3_Se_2_, Mn_4_Se_3_, Mn_2_Se_2_, Mn_3_Se_4_, Mn_2_Se_3_, and MnSe_2_. The Se/[Mn + Se] ratio is thus varied from 0.29 to 0.67. The atomic structures for all of the lowest energy phases are shown in Figure S7.

**Figure 3:**
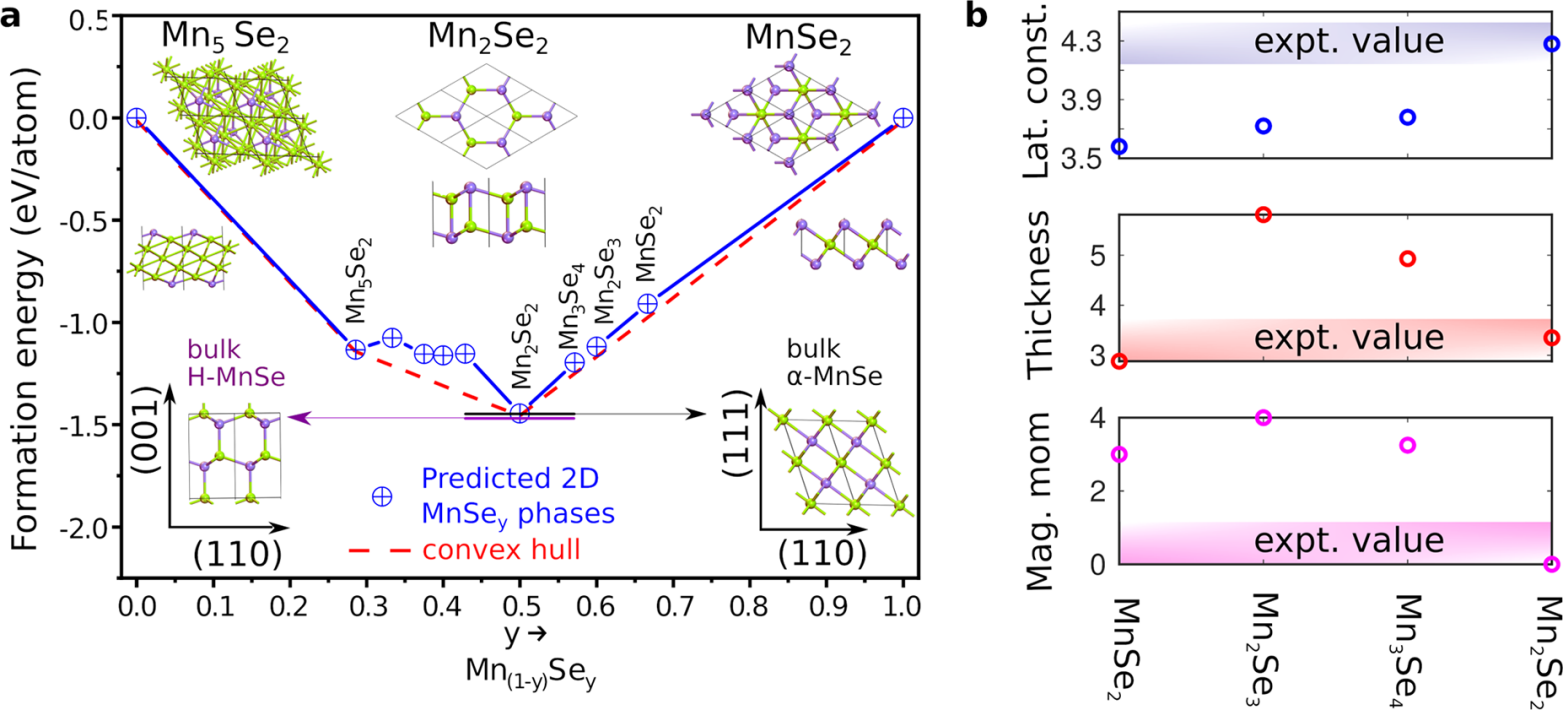
(a) PBE+U calculated 2D Mn–Se phase diagram and the atomic structures for the lowest energy phases are shown as insets. (b) Material parameters for the stable candidates with hexagonal symmetry.

To construct the phase diagram, we have calculated the formation energy per atom for all of the lowest energy structures obtained from USPEX calculations. The evolution of the energies during the optimization procedure are shown in Figure S8. All USPEX DFT calculations were done within normal PBE exchange–correlation functional. The formation energies of transition metal oxides and chalcogenides are known to be underestimated by PBE functional,^34^ and thus we recalculated the lowest energy phases using PBE+U (*U* = 2.3 eV applied to Mn d-orbitals). PBE+U accounts for the strong on-site electron correlations better and should also improve lattice constants and magnetic properties. The resulting 2D Mn–Se phase diagram and the atomic structures for selected phases are shown in Figure 3a. The full list of properties of all stable phases are given in Table S4. The convex hull is determined by two phases, Mn_5_Se_2_ and Mn_2_Se_2_. The structures for MnSe_2_, Mn_2_Se_3_, and Mn_3_Se_4_, *etc.*, are obtained when the bulk α-MnSe is thinned down to the atomic level with a Se-terminated surface. The formation energies of all of these phases are very close to the convex-hull line, and the formation energy of bulk α-MnSe is nearly degenerate with Mn_2_Se_2_ phase. We note that the results are somewhat sensitive to the choice of the +U parameter (*cf.* the formation energies of two- and three-dimensional MnSe structures in Figure S9). For instance, with PBE the square phase (FeSe-type structure) of Mn_2_Se_2_ is lower in energy than the hexagonal phase. When the U parameter is introduced, the hexagonal phase becomes more stable than the square phase. For *U* > 2 eV, the bulk structures (α-MnSe and SiC-like H-MnSe) tend to become more stable than the isolated two-dimensional structures. For *U* = 2.3 eV (the value used in the following), bulk rock-salt-like α-MnSe structure is energetically nearly degenerate with the hexagonal Mn_2_Se_2_ phase, while the SiC-like bulk phase is 20 meV/atom lower in energy. When the binding between the 2D sheet and the substrate is accounted for, however, the 2D Mn_2_Se_2_ becomes again the lowest energy phase.

Using the collected set of stable phases close to the convex hull, we can further narrow down the candidates. From experiments we know that the surface displays a triangular lattice, the lattice constant is about 4.3 Å, and the layer is not ferromagnetic. The only phases that would lead to a triangular lattice in STM images are Mn_2_Se_2_ and the α-MnSe derivatives. Their lattice constants, thicknesses, and magnetic moments are collected in Figure 3b. On the basis of this, Mn_2_Se_2_ is clearly the only structure that both is stable and shows the lattice constant, lattice symmetry, and thickness in agreement with the STM results. The experiments indicated that the layers are not ferromagnetic. α-MnSe derivatives are calculated to be ferromagnetic (see the energy differences between FM and AFM states in Table S5), which agree with previous predictions^14,26^ and possibly also experimentally verified in ref (8). Among these candidates, only Mn_2_Se_2_ is calculated to be antiferromagnetic and semiconducting, in agreement with the experimental findings. Mn_2_Se_2_ is also dynamically stable, as shown by the calculated phonon dispersion curves in Figure S10. Thus, by combining the experimental and computational results, we arrive at the conclusion that the observed phase corresponds to Mn_2_Se_2_, which is antiferromagnetic and a semiconductor.

The atomic structure of the MnSe phase (now dropping the redundant subscripts) is shown in Figure 3a. The structure resembles that of bilayer h-BN but with stronger interlayer interactions and buckling in the two sublattice sites reminiscent of silicene or the predicted 2D III–V phases.^35,36^ We note that Mn–Se bonds in all discovered MnSe_*x*_ phases are close to 2.6 Å. Consequently, the only way to obtain hexagonal phases with lattice constants as large as 4.3 Å is when the bonds are nearly in-plane (√3 × 2.6 Å = 4.5 Å). Layers with a similar structure can be found in bulk β-CuI,^37^ but, on the basis of our database and literature searches, we found no synthesized or exfoliated 2D materials with this structure.

**Figure 4:**
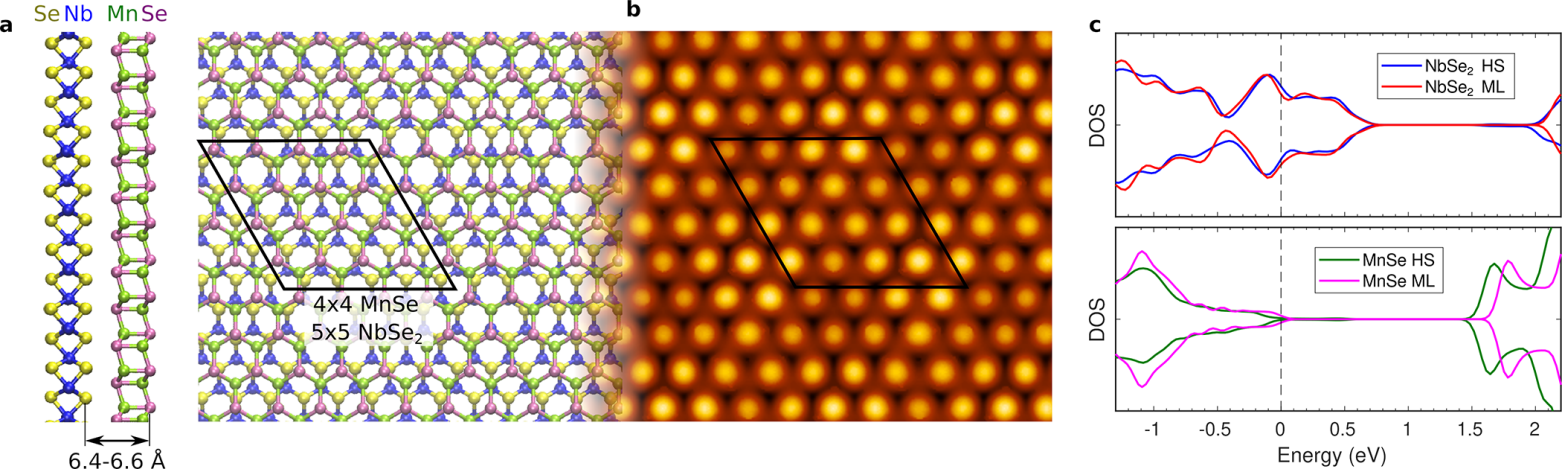
(a) Side and top views of the atomic structure of the NbSe_2_/MnSe heterostructure. The supercell is indicated by a black rhombus. (b) Simulated STM image for pristine heterostructure surface. (c) DOS from isolated monolayers (MLs) and the corresponding local DOS from the layers in the heterostructure (HS). Energy zero is set at the Fermi level and at valence band maximum in the case of MnSe monolayer.

With a likely structural candidate at hand, we then tried to reproduce the experimental STM images, which revealed a moiré pattern with 4 × 4 periodicity, and also a large concentration of defects, mostly located at the same point of the moiré pattern. Using the calculated lattice constants of 3.435 Å for NbSe_2_ and 4.229 Å for MnSe, we find that 5 units of NbSe_2_ indeed nearly matches 4 units of MnSe. We thus constructed a model for the NbSe_2_/MnSe heterostructure using a 4 × 4 supercell of MnSe placed on top of a 5 × 5 supercell of NbSe_2_; see atomic structure in Figure 4a. The MnSe layer is unstrained, and the NbSe_2_ layer is under 1.5% compressive strain. The layers are bound together by van der Waals forces, as indicated by the small corrugation across the moiré and the calculated interlayer binding energy of 25 meV/Å^2^, which is similar to the values found in other layered materials.^38^ The distance of about 6.5 Å from the top Se layer of NbSe_2_ to the top Se layer of MnSe matches well with the apparent layer height in STM; *cf.*Figure 1.

The simulated STM image from the heterostructure is shown in Figure 4b and shows a moiré pattern similar to the experimental one shown in Figure 1c, although with a smaller contrast. The contrast difference may arise from the strain of NbSe_2_ in our calculations or from the fact that the substrate in our model consists of only a monolayer NbSe_2_. As expected, the bright protrusions originate from the top layer Se atoms. In order to verify that the computational results are also consistent with the STS spectra, we show the density of states of the MnSe monolayer and the local density of states from the heterostructure in Figure 4c. The simulated STS spectra for the isolated MnSe and NbSe_2_ are shown in Figure S11. Upon joining the MnSe and NbSe_2_ layers, the valence band maximum of MnSe is 0.1–0.2 eV below the Fermi level, while the NbSe_2_ states are hardly affected due to the much higher DOS at the Fermi level. The VBM falling very close to the Fermi level in the heterostructure, the intense feature at about −1 eV, and band gap larger than 1.5 eV all match well with the experimental findings (given the usual band gap underestimation in DFT calculations). The shift of the NbSe_2_ Nb d-band derived peak from about 0.4 to 0.3 V in the experimental spectra is not reproduced in our calculations for the pristine heterostructure, although subject to sample-to-sample variations (*e.g.*, a shift of about 40 mV in Figure S4).

**Figure 5:**
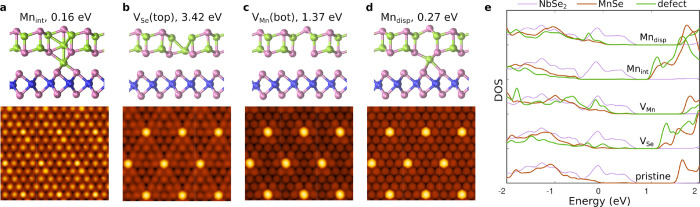
(a–d) Atomic structures, formation energies, and the simulated STM images of the various defect candidates. (e) Local density of states in the two monolayers and at the defect (including defect and first nearest neighbor sites) for the pristine system and for the defects in panels a–d. Energy zero is at the Fermi level.

In order to identify the most likely defect causing the bright protrusions in the experimental images, we first calculated the formation energies of a set of native defects in the heterostructure system. We narrowed down the candidates to those shown in Figure 5a–d based on the following reasoning. In experiments, the protrusions were always located in the Se-site and thus the corresponding defect could be either a Se vacancy on top or a Mn vacancy on bottom. The simulated STM images for both of these vacancies agree well with the experimental image, and thus they are viable candidates. According to calculations, both Mn and Se adatoms prefer to locate in the hollow (center-of-hexagon) site and can thus be ruled out. Mn_Se_ antisite is located in the correct site but has a very large formation energy. The simulated STM images for these unlikely defects are shown in Figure S12. The Mn interstitial located in certain positions between NbSe_2_ and Mn_2_Se_2_ shows strikingly low formation energy. However, the simulated STM image appears nearly indistinguishable from the pristine surface results, and thus Mn interstitials cannot be the origin of the bright protrusions. On the other hand, they modulate the interlayer separation and thus could contribute to the intensity variations across the surface. It is also worth noting that, at increased Mn interstitial concentrations, the average formation energy becomes fairly large (more than 1 eV) and thus there should be only a moderate concentration of interstitials present.

In addition to these ”obvious” defect candidates, we also found an unusual defect, where the Mn is displaced from the MnSe layer to the interstitial site (Mn_disp_). It could also be considered a pair of interstitial and vacancy in the nearest neighbor sites but with formation energy much lower than the sum of the isolated constituents. Likely reasons for the low formation energy are that it forms locally a wurtzite-like structure similar to the H-MnSe phase in Figure 3a and it interacts with the NbSe_2_ substrate. Similar to the Mn interstitial, at larger concentration of these defects the formation energies increase markedly, and when we attempted to place wurtzite-like 2D-Mn_2_Se_2_ on NbSe_2_, it spontaneously transformed to our proposed Mn_2_Se_2_, except for few sites in the moiré pattern. Interestingly, this defect is unstable in freestanding monolayer MnSe as it requires interaction with the substrate Se atoms. The simulated STM image shows again a bright protrusion in good agreement with experimental images.

The local DOS of V_Se_ and Mn_disp_ shown in Figure 5e remain very similar to the pristine one, and the main peak remains at −1 eV as also observed in the experiments. In contrast, V_Mn_ leads to a shift to higher energies and Mn_int_ shifts to lower energies, both of which seem inconsistent with experimental spectra. In the end, although three defects (V_Se_, V_Mn_, and Mn_disp_) produced STM images in agreement with the experimental ones, and V_Se_ and Mn_disp_ are also consistent with STS, we assign these protrusions to Mn_disp_ defect on the basis of its significantly lower formation energy compared to that of V_Se_.

**Figure 6:**
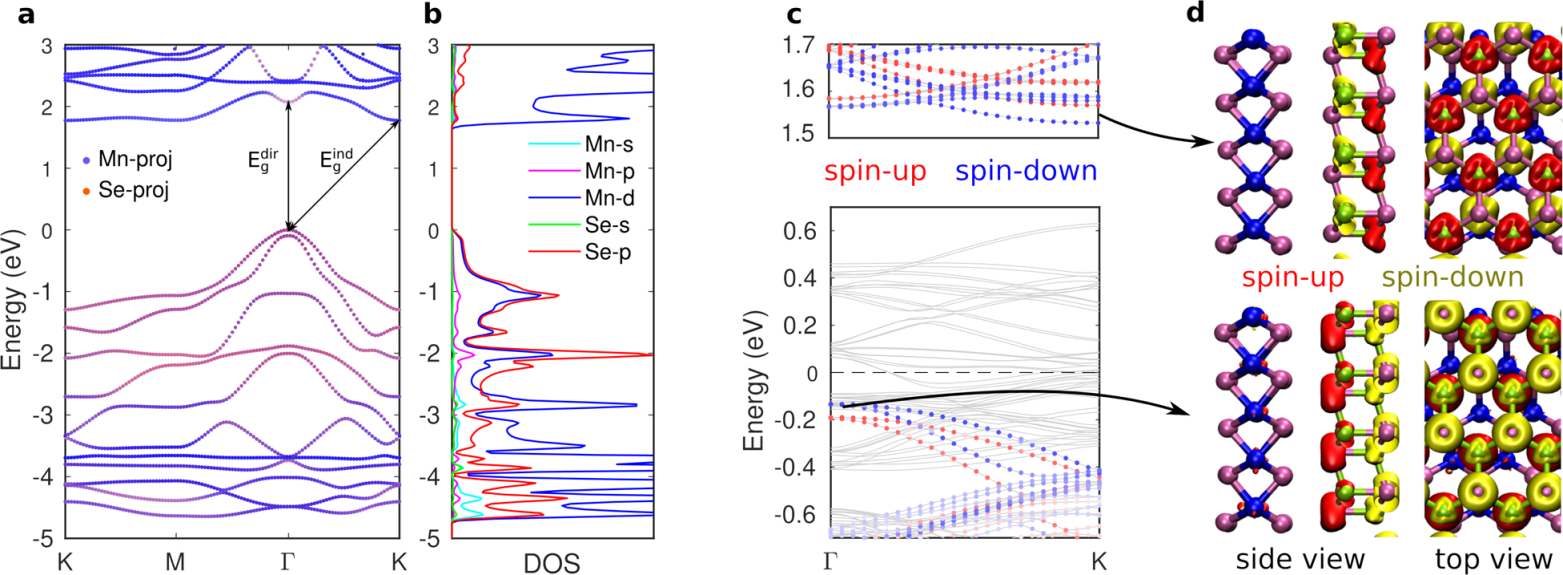
Atomic and electronic structures of Mn_2_Se_2_. (a) Band structure with the color indicating the state projection to Mn (blue) and Se (red) atoms and (b) the corresponding projected density of states. The minimum direct and indirect band gaps are also shown. (c) Band structure of the NbSe_2_/MnSe heterostructure (gray lines). The spin-up (red) and spin-down (blue) states of MnSe layer are highlighted. (d) Spin-density isosurfaces from the VBM and CBM states of MnSe.

Having identified the structure of the MnSe phase, we now focus more on its properties. Band structure and density of states projected on the Mn and Se atoms calculated using PBE+U and accounting for the spin–orbit coupling are shown in Figure 6a,b. MnSe is an indirect gap semiconductor with a sizable band gap of 1.83 eV. VBM is located at the Γ-point and CBM at the K-point with a very flat band between K- and M-points (and nearly degenerate valley minima), resulting in a very large electron effective mass in that direction. The minimum direct gap at the Γ-point is 2.07 eV with large oscillator strength (Figure S13) and a corresponding peak in the absorption spectrum. We expect that upon absorption, the electrons at CBM will rapidly decay to K-/M-points and result in momentum indirect excitons. The flatness of the lowest conduction band along K–M could have interesting consequences on the exciton structure.

As mentioned above, MnSe is antiferromagnetic, but since the opposite magnetic moments (±4.4 μ_B_ from PBE+U calculations) are located in the two Mn layers, it also corresponds to two ferromagnetic planes contained within a monolayer of the material. The antiferromagnetic state is 0.23 eV lower (per Mn atom) than the fully ferromagnetic state (and several eV lower than the nonmagnetic state; see Table S5 for details), thus demonstrating that the AFM state is highly robust. On the basis of literature searching and querying the 2D materials database,^15,39,40^ we found only one synthesized 2D material with similar antiferromagnetic ordering: Mn_3_N_2_, which was recently synthesized using a salt-templated growth in solution.^41^ Such ordering could be very promising for spintronic applications, such as spin filters, since the spin degeneracy can be lifted by, *e.g.*, electric potential varying in the out-of-plane direction (Figure S14). Curiously, we found that a similar effect is already taking place in our NbSe_2_/MnSe heterostructure, where the interaction of the MnSe with NbSe_2_ on one side leads to lifting of the spin degeneracy. As shown in Figure 6c, the states corresponding to MnSe VBM and CBM can be found among the (folded) bands of the heterostructure and show a strong splitting for the spin-up and -down states of 60 meV in the VBM (Γ-point) and 40 meV in the CBM (K-point). As a result, both the VBM and CBM are spin-down, but the VBM state is localized to the top Mn atoms whereas the CBM state is localized to the bottom Mn atoms.

## Conclusions

We have reported on the successful synthesis of monolayer MnSe on NbSe_2_ substrate. The material is characterized and identified using STM and STS experiments and global structure prediction at the density functional theory level and found to possess an unusual atomic structure similar to that found in the layered bulk phase of CuI or in the bilayer of *h*-BN but with buckling. Although the number of transition metal chalcogenide materials is large, the number of structural prototypes is relatively small, and thus the phase reported here expands not only the library of known synthesized 2D materials but also the library of prototypes. Finally, calculations revealed this material to exhibit out-of-plane antiferromagnetic ordering that leads to two ferromagnetic planes within a single sheet of material.

## Methods

### Experimental Methods

#### Sample Growth

The MnSe monolayer was grown on bulk NbSe_2_ substrate by e-beam evaporation of Mn and co-deposition of Se from a Knudsen cell in UHV conditions with a base pressure ∼ 10^–10^ mbar. Before growth of the MnSe, the bulk NbSe_2_ was cleaved inside the vacuum chamber and degassed at around 600 K temperature for 30 min. During the growth of MnSe, the substrate was kept at 490 K temperature. The Mn flux (10 nA) and Se flux, with equivalent pressure ∼ 10^–8^ mbar, were directed to the surface of the NbSe_2_ substrate. The growth of the MnSe is dictated by the Mn flux, and the excess selenium desorbs from the substrate since the substrate temperature was higher than the evaporation temperature of selenium atoms (*T* = 393 K). Temperatures were measured with an optical pyrometer (Metis MP25, SensorTherm GmbH) directly from the sample surface. Depending on the MnSe coverage, the Mn is deposited for 10–20 min followed by annealing of the sample for another 20 min in a Se-rich environment. The samples were characterized *in situ* by STM. The growth parameters to obtain MnSe are listed in Table S1. For some samples we observed intercalation patterns in the substrate which retained the CDW; see Figure S3 for details. Growth of MnSe on HOPG, however, was unsuccessful; see Table S2 for the list of attempted growth parameters.

#### Sample Characterization

After the sample growth, it was inserted into a low-temperature STM (Unisoku USM-1300) connected to the sample preparation chamber. The subsequent measurements were performed at *T* = 4 K or 350 mK. STM images were taken in the constant current mode. d*I*/d*V* spectra were recorded by standard lock-in detection while sweeping the sample bias in an open feedback loop configuration, with a peak-to-peak bias modulation of 5 mV (long-range spectra) or 0.2 mV (short-range spectra of the superconducting gap) at a frequency of 709 Hz. In subsequent measurements, X-ray photoemission spectroscopy was performed with a PHOIBOS 1D 100 DLD (SPECS GmbH) instrument in the same UHV system as the STM (Createc LT-STM).

### Computational Methods

In order to reveal the right composition and the exact atomic structure of the synthesized Mn–Se compound, we have used USPEX code which is successful in crystal structure prediction for 1D, 2D, and 3D structures.^33^ Here, we have studied 2D structures for Mn_5_Se_2_, Mn_4_Se_2_, Mn_5_Se_3_, Mn_4_Se_3_, Mn_3_Se_2_, Mn_2_Se_2_, and Mn_2_Se_3_. The maximum thickness of the 2D layer is set to be 7.5 Å. In the crystal structure prediction process, the population size for every generation is 20. In the first generation, all of the structures are randomly generated with a randomly selected space group of 2D layers. From the second generation onward, we generate 30% of structures by heredity, 40% of structures by random symmetric structure generator, 10% of structures by permutation, 10% of structures by lattice mutation, and 10% by atomic mutation (also known as soft mutation or coormutation). A maximum of 80 generations are created unless a same structure is retained as best for 20 generation. From the model calculation of Mn_1_Se_1_ in hexagonal boron nitride structure, it is understood that the observed lattice parameter of 4.3 Å is achievable when MnSe atoms are in-plane-bonded in the BN structure. On the other hand the measured height of the MnSe island shows that there must be minimum two atomic layers in the 2D layer. Thus, we also studied the hexagonal Mn_2_Se_2_ structure for reference. Similarly, MnSe_2_ is known to be stable in tetragonal phase, so it has also been considered in the convex-hull construction.

All of the structures generated from USPEX code are studied by density functional theory calculations using Vienna *Ab Initio* Simulation Package (VASP).^42,43^ Generalized Gradient Approximation (GGA) proposed by Perdew, Burke, and Ernzerhof (PBE) was used to correct the exchange and correlation energies in DFT calculations.^44^ Lattice parameters of all structures, for *a* and *b* axes, are relaxed by keeping the total volume of the cell constant. Electronic relaxations are carried out with the convergence condition of 10^–5^ eV. Ionic relaxations are carried out until the absolute forces on each atom becomes less than 0.01 eV/Å. The reciprocal-space resolution for *k*-point mesh generation is set to be 0.05 2π/Å. It is known that GGA fails to predict the electronic properties of Mn compounds. Thus, we carried out GGA+U calculations with a *U* value of 2.3 eV set for all Mn atoms in the lowest energy structures. The *U* value for Mn-site is chosen to match the optimized lattice parameters obtained from the HSE06 calculation on the hexagonal Mn_2_Se_2_ layer.^45,46^ The adopted value is also close to the *U* = 2 eV value for α-MnSe and zinc-blende MnSe found by Amiri *et al.* and Zhou *et al.* following careful benchmarking of the effects of *U* on the structural, electronic, and magnetic properties.^47,48^ Further, the *U* values are varied to understand the relative stability between hexagonal and square Mn_2_Se_2_ layers. Without the *U* value, the square lattice was found to be stable, while for *U* = 2.3 eV both structures are energetically degenerate with negligible energy difference.

Formation energy is defined as
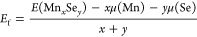
1where *E*(Mn_*x*_Se_*y*_) and μ(Mn) and μ(Se) are the total energies of the Mn_*x*_Se_*y*_ layer and chemical potentials of Mn and Se atoms, calculated from bulk α-Mn and bulk Se. Since the synthesis took place under excess selenium, defect formation energies are calculated at the Se-rich limit (μ_Se_ from the Se bulk phase and μ_Mn_ = *E*(MnSe – μ_Se_).

The heterostructure of MnSe and NbSe_2_ is constructed using an unstrained 4 × 4 supercell of MnSe and a 5 × 5 supercell of NbSe_2_ under 1.5% compressive strain. These calculations are carried out with GGA+U(2.3), as justified above, but also accounting for the van der Waals forces *via* the semiempirical-*D*3 corrections.^49^ The Brillouin zone is sampled using 2 × 2 *k*-point mesh. STM images are simulated *via* Tersoff–Hamann approximation using p4vasp software.

## Supporting Information Available

Additional details of the synthesis and experimental characterization results; further information about the different structures found by USPEX; additional calculations for the material properties of MnSe (PDF)
