# Supplementary material for: Synthesis and Properties of Monolayer MnSe with Unusual Atomic Structure and Antiferromagnetic Ordering
Source: ACS Nano. 2021 Jul 27;15(8):13794–802. doi: 10.1021/acsnano.1c05532 (PMC8388122; doi:10.1021/acsnano.1c05532)
Supplement: Supplementary file 1 — nn1c05532_si_001.pdf [file nn1c05532_si_001.pdf]

Supporting information to "Synthesis and properties of monolayer MnSe with unusual atomic structure and antiferromagnetic ordering"

Markus Aapro,[†,§] Md Nurul Huda,[†,§] Jeyakumar Karthikeyan,[†,‡] Shawulienu Kezilebieke,[†] Somesh C. Ganguli,[†] Héctor González Herrero,[†] Xin Huang,[†] Peter Liljeroth,[†] and Hannu-Pekka Komsa[*,†,¶]

†*Department of Applied Physics, Aalto University, 00076 Aalto, Finland*

‡*Rajiv Gandhi Institute of Petroleum Technology, Jais, Amethi - 229304, Uttar Pradesh, India*

¶*Microelectronics Research Unit, University of Oulu, 90014 Oulu, Finland*

§*Contributed equally to this work.*

E-mail: hannu-pekka.komsa@oulu.fi

Moiré pattern Fourier analysis

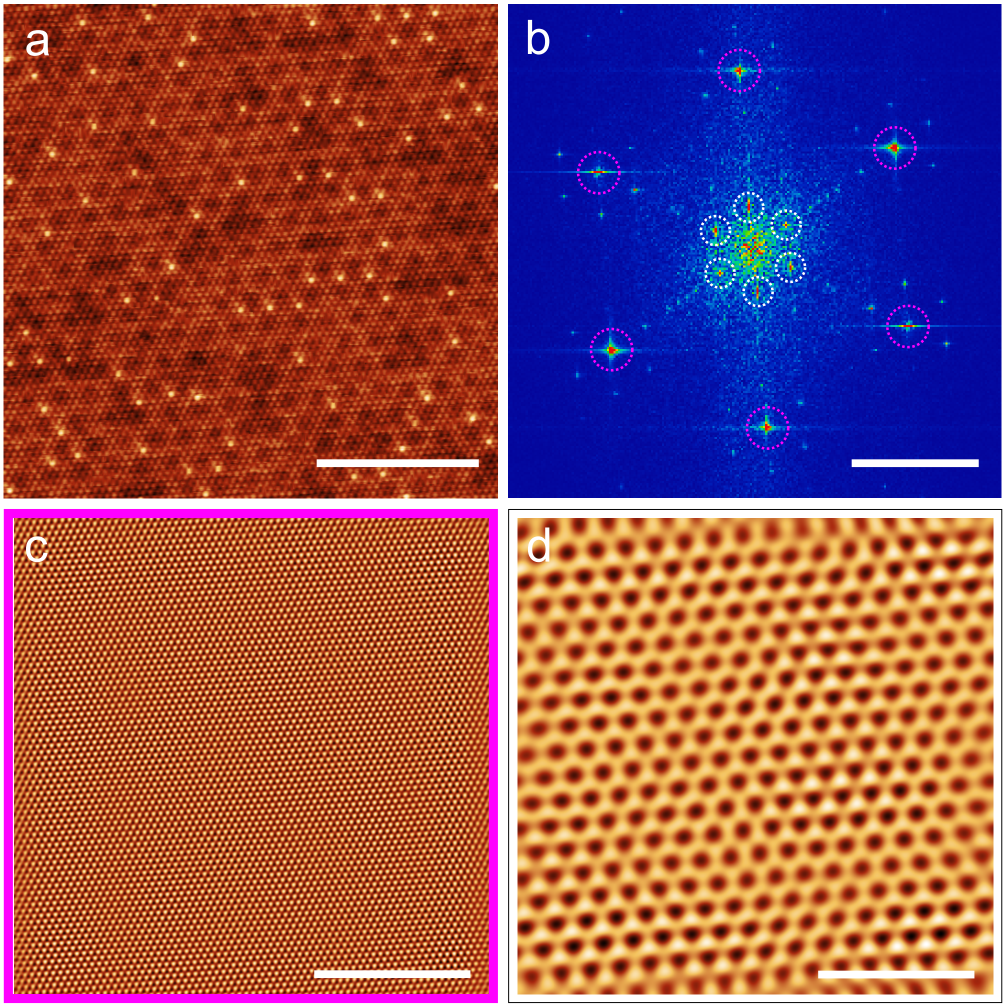

Figure S1: (a) STM image on a MnSe island. $V_{bias} = -0.1$ V, I = 200 pA, scale bar 10 nm. (b) Fast Fourier transform of (a), with moiré and Bragg peaks highlighted with white and magenta, respectively. Scale bar 2 nm^{-1}. (c) Inverse Fourier transform of the Bragg peaks, with the lattice corresponding to the Se lattice. Scale bar 10 nm. (d) Inverse Fourier transform of the moiré peaks. Scale bar 10 nm.

Abrikosov vortices in $Mn_2Se_2/NbSe_2$ heterostructures

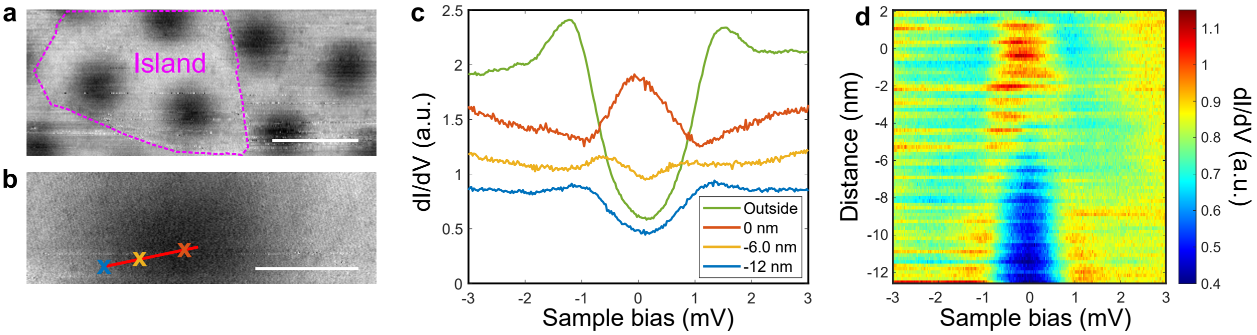

Figure S2: Vortex spectroscopy. (a) dI/dV map showing the Abrikosov vortex lattice. An MnSe island is highlighted in fuchsia. Map measured in the constant-current mode with $V_{bias} = 1.5$ mV, I = 50 pA, modulation voltage 150 μV, scale bar 50 nm. (b) An individual vortex. The red line shows the line spectrum in (d), with the spectra in (c) highlighted with crosses. $V_{bias} = 1.5$ mV, I = 500 pA, modulation voltage 150 μV, scale bar 15 nm. (c) Spectra at various distances from the vortex core. The background spectrum is an average of 70 points over a 12.7 nm distance, while the other spectra are averages of 5 spectra over a 0.9 nm distance. Voltage modulation 0.2 mV. (d) Line spectra along the red line shown in (b), voltage modulation 0.2 mV.

Growth parameters on $NbSe_2$ substrate

Table S1: Growth parameters used to obtain the MnSe islands, where T_s is the substrate temperature, t_d is the deposition time, I_{Mn} is the Mn flux as measured by a Focus EFM 3 e-beam evaporator, p_d is the deposition pressure, T_a is the post-annealing temperature and t_a is the post-annealing time. Samples C and D were grown in a different vacuum chamber from A and B.

Sample	T_s (°C)	t_d (min)	I_{Mn} (nA)	p_d (mbar)	T_a (°C)	t_a (min)
A (Fig 2)	215	5	10	$3.0 \cdot 10^{-8}$	220	21
B (Fig 1)	215	5	5	$2.0 \cdot 10^{-8}$	215	16
C (Fig S5)	210	10	10	$5.3 \cdot 10^{-8}$	210	12
D (Fig S4)	220	10	10	$5.1 \cdot 10^{-8}$	220	11

Unsuccessful growth parameters on HOPG substrate

Table S2: Growth parameters used in unsuccessful attempts to grow MnSe islands on HOPG. T_s is the substrate temperature, t_d is the deposition time, I_{Mn} is the Mn flux as measured by a Focus EFM 3 e-beam evaporator, p_d is the deposition pressure, T_a is the post-annealing temperature and t_a is the post-annealing time.

T_s (°C)	t_d (min)	I_{Mn} (nA)	p_d (mbar)	T_a (°C)	t_a (min)
300	5	3.40	$1.10{\cdot}10^{-8}$	300	20
300	10	5.50	$2.00{\cdot}10^{-8}$	300	20
300	5	5.00	$2.00{\cdot}10^{-8}$	300	5
250	5	5.00	$1.00{\cdot}10^{-8}$	250	3
200	5	5.00	$1.00{\cdot}10^{-8}$	200	3
240	5	10.00	$2.00{\cdot}10^{-8}$	240	1.5
180	5	10.00	$2.40{\cdot}10^{-8}$	180	1
180	15	10.00	$1.80{\cdot}10^{-8}$	180	1
20	10	10.00	$2.20{\cdot}10^{-8}$		0
216	10	10.00	$2.20{\cdot}10^{-8}$	216	10
300	5	10.00	$2.40{\cdot}10^{-8}$		0
220	5	10.00	$2.40{\cdot}10^{-8}$	220	10

Effect of Mn intercalation on the $NbSe_2$ substrate

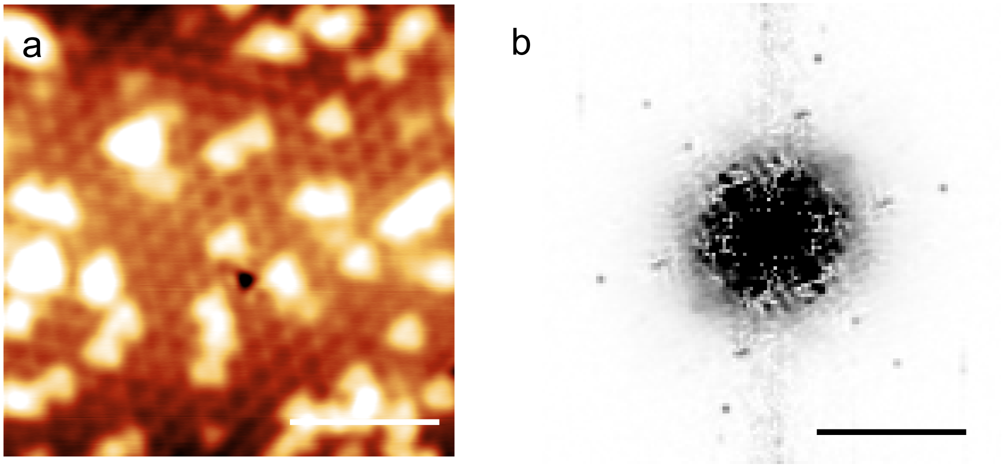

Figure S3: (a) Atomic resolution STM scan and (b) the corresponding 2D FFT pattern of the $NbSe_2$ substrate in sample C (as defined in Table S1). The intercalation does not significantly alter the electronic structure of the substrate, as seen in the unperturbed CDW. $V_{\mathrm{bias}} = 0.1$ V, $I = 200$ pA, scale bar 5 nm in a) and 3 nm^{-1} in b).

Repeatability of growth process

To verify repeatability of our growth process, we successfully synthesized more samples in another vacuum chamber and studied them with LT-STM (Createc GmbH) and XPS (PHOIBOS 1D 100 DLD, SPECS GmbH). Results are collected in Fig. S4. The samples exhibited the same properties as the previous ones in terms of lattice constants, high contrast impurities and electronic properties as probed by STS. With this we conclude that our synthesis is repeatable and reasonably robust in terms of growth parameters.

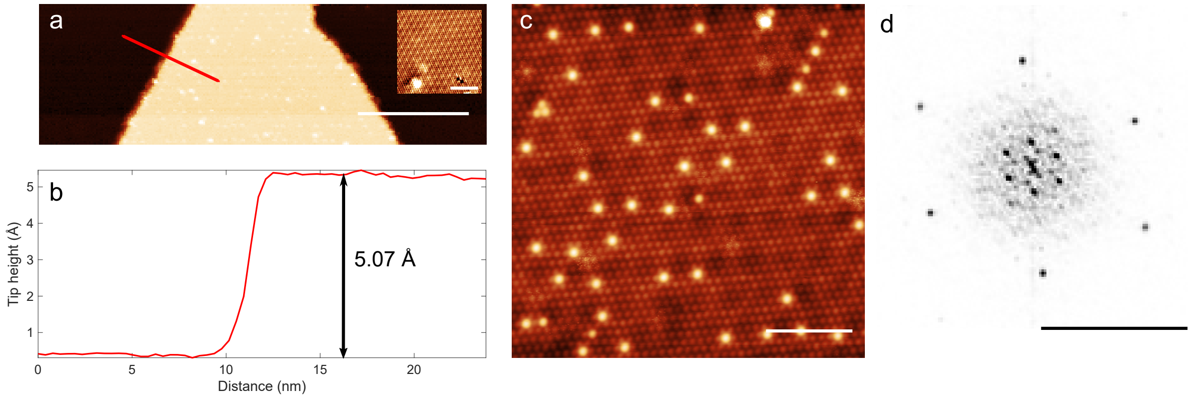

Figure S4: (a) Large area STM scan showing MnSe islands on $NbSe_2$ substrate. $V_{\text{bias}} = 1.059$ V, $I = 2.42$ pA, scale bar 25 nm. Insert: atomic resolution scan of the $NbSe_2$ substrate. $V_{\text{bias}} = -58.7$ mV, $I = 168.2$ pA, scale bar 5 nm. (b) Line profile along the red line shown in (a). (c,d) Atomic resolution scan (c) and the corresponding 2D-FFT (d) on a MnSe island. $V_{\text{bias}} = 378$ mV, $I = 33$ pA, scale bars 5 nm and 5 nm^{-1} for c) and d), respectively.

Additional line spectra over the island edge

A line spectra at extended bias range is shown in Fig. S5. In Fig. S5(b), we observe the MnSe conduction band onset at $1.8 - 2$ eV. We also observe periodic modulations on the MnSe islands in the line spectra shown in figure S5(a,d). As the features in lower energies are also modulated with the same energy amplitude, we interpret this as local electrostatic potential and possibly interlayer separation modulation caused by the moiré pattern.

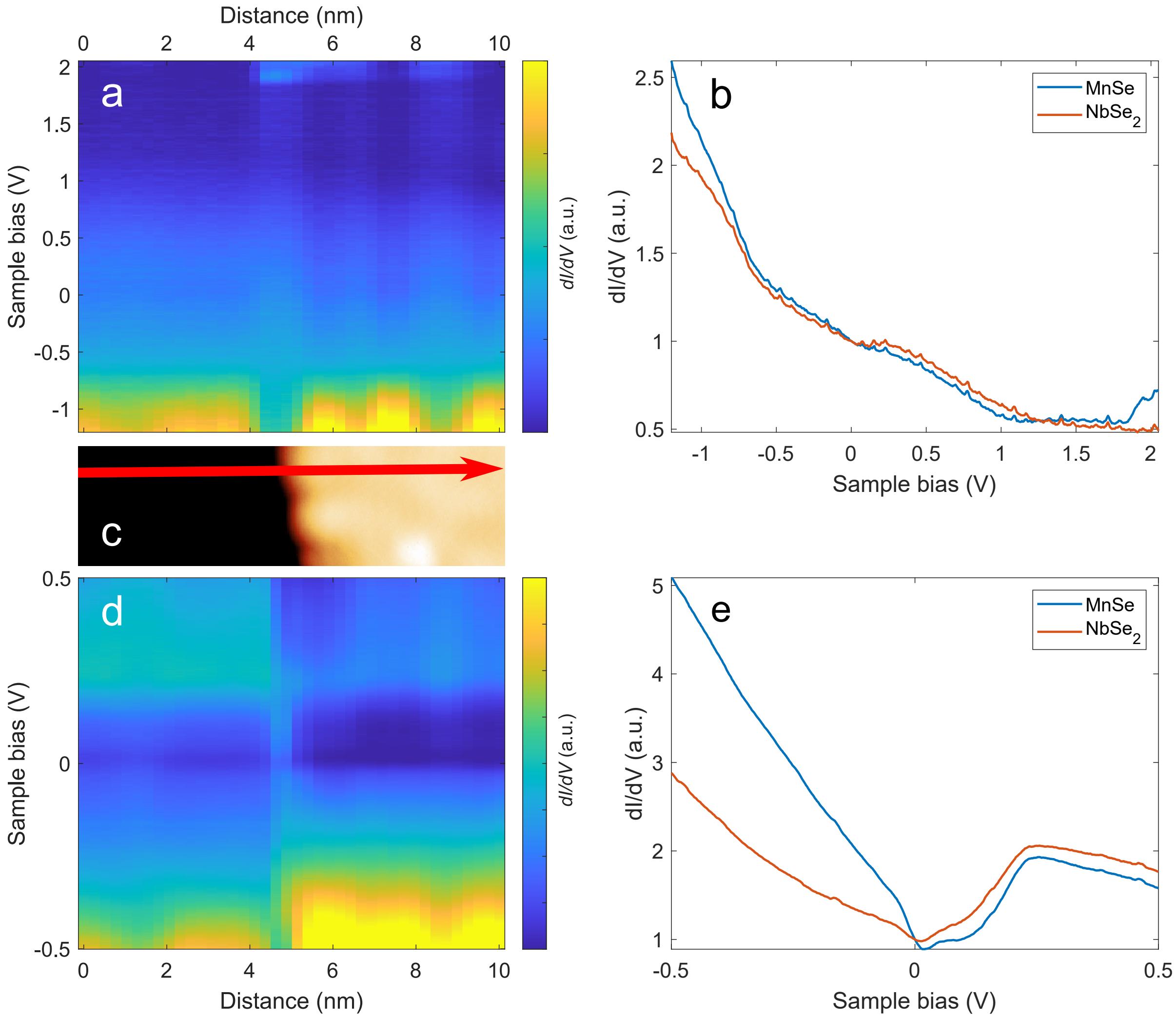

Figure S5: Line spectra across a MnSe island edge on $NbSe_2$. (a,b) Heat map and layer averages of large energy range line spectra taken across the red arrow in panel c. Voltage modulation 20 mV. (c) Topography scan of the island edge. Sample bias 1 V, current 5 pA. (d,e) Short range line spectra and layer averages taken along the same trajectory. The d-band shifts by approximately 37 mV on the MnSe as opposed to the 96 mV shift in Fig 2a.

Photoemission spectra before and after N_2 exposure

Effects of nitrogen gas exposure were studied by XPS (PHOIBOS 1D 100 DLD, SPECS GmbH) and STM. The MnSe/$NbSe_2$ sample (D in table S1) was exposed to pressures between $1 - 1000$ mbar for 5 minutes. It should be noted that the analysed spot size was larger than the sample surface, resulting in a background signal from the sample holder.

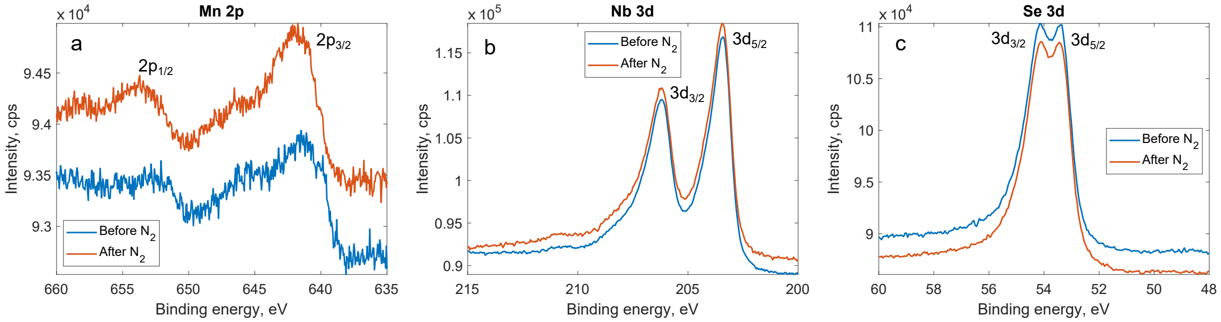

Figure S6: (a) High-resolution XPS spectrum of the Mn 3d region before and after exposure to N_2, showing a shift in the $2p_{1/2}$ and $2p_{3/2}$ peaks. (b) The Nb 3d region. (c) The Se 3d region showing a distinct splitting of the $3d_{5/2}$ and $3d_{3/2}$ peaks. Energy scales were calibrated by setting the Nb $3d_{5/2}$ peaks to literature value (203.4 eV).

Figure S6 shows high-resolution photoemission spectra for Mn, Se and Nb transitions. An energy shift is observed in the Mn peaks, whereas the Nb and Se peaks remain intact. This suggests that the substrate surface remains chemically intact besides the Mn compounds: due to the low coverage of MnSe on the sample, it is conceivable that the Se peaks remain indistinguishable even if the MnSe undergoes a reaction.

The atomic concentrations were calculated by correcting the XPS peak intensities with a transmission function using the software CasaXPS. Due to the spot size the MnSe island stoichiometry cannot be accurately determined from this measurement. Regardless, the concentrations remaining mostly intact suggest that the N_2 exposure did not destroy the substrate or remove material from the surface.

Table S3: Calculated atomic concentrations based on high-resolution XPS spectra on the Mn 2p, Se 3d, Nb 3d and C 1s energy regions. The errors are on the order of 5% of values given.

Sample	Mn (at.-%)	Se (at.-%)	Nb(at.-%)	C (at.-%)
Before N_2 exposure	1.3	38.3	18.4	42.0
After N_2 exposure	1.6	38.1	19.4	40.9

We did not observe any MnSe islands on the exposed sample in the STM. While we cannot definitively exclude the possibility of some islands surviving the exposure, it caused the sample condition to degrade markedly. As such, we do not expect the MnSe layers to be

air stable.

Additional computational data

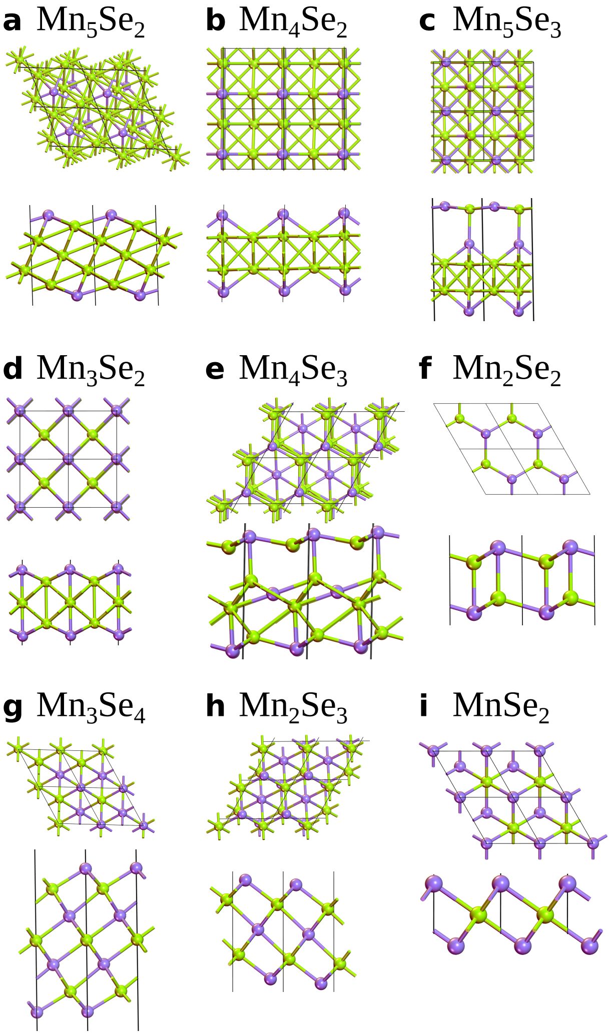

Figure S7: Top and Side views of (a) Mn_5Se_2, (b) Mn_4Se_2, (c) Mn_5Se_3, (d) Mn_3Se_2, (e) Mn_4Se_3, (f) Mn_2Se_2, (g) Mn_3Se_4, (h) Mn_2Se_3, and (i) $MnSe_2$ layers. Here, chartreuse and lavender color balls represent Mn and Se atoms, respectively and the unit-cell are shown in black lines. 2×2 supercells are shown for all structures.

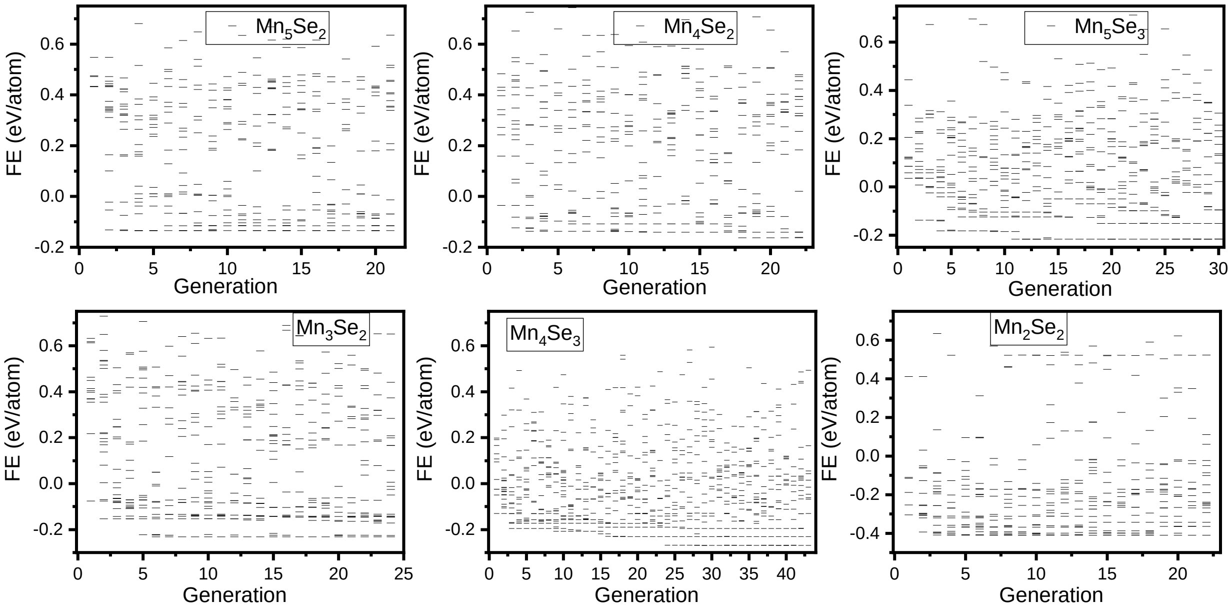

Figure S8: Formation energies (FE) from the USPEX runs as a function of the generation.

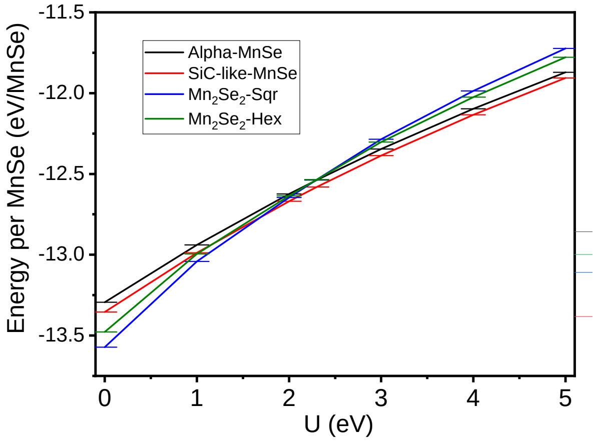

Figure S9: Energy per MnSe formula unit is plotted against Hubbard U value.

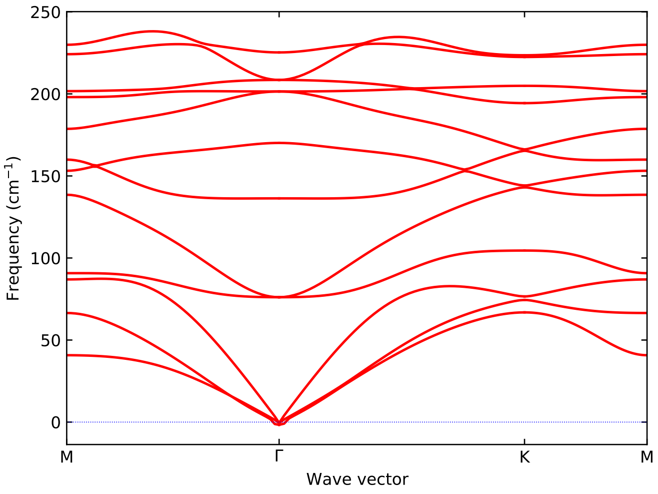

Figure S10: Mn_2Se_2 phonon dispersion curves calculated using PBE+U(2.3) and 4×4 supercell. There are 3E+3A_1 optical modes at the Γ-point.

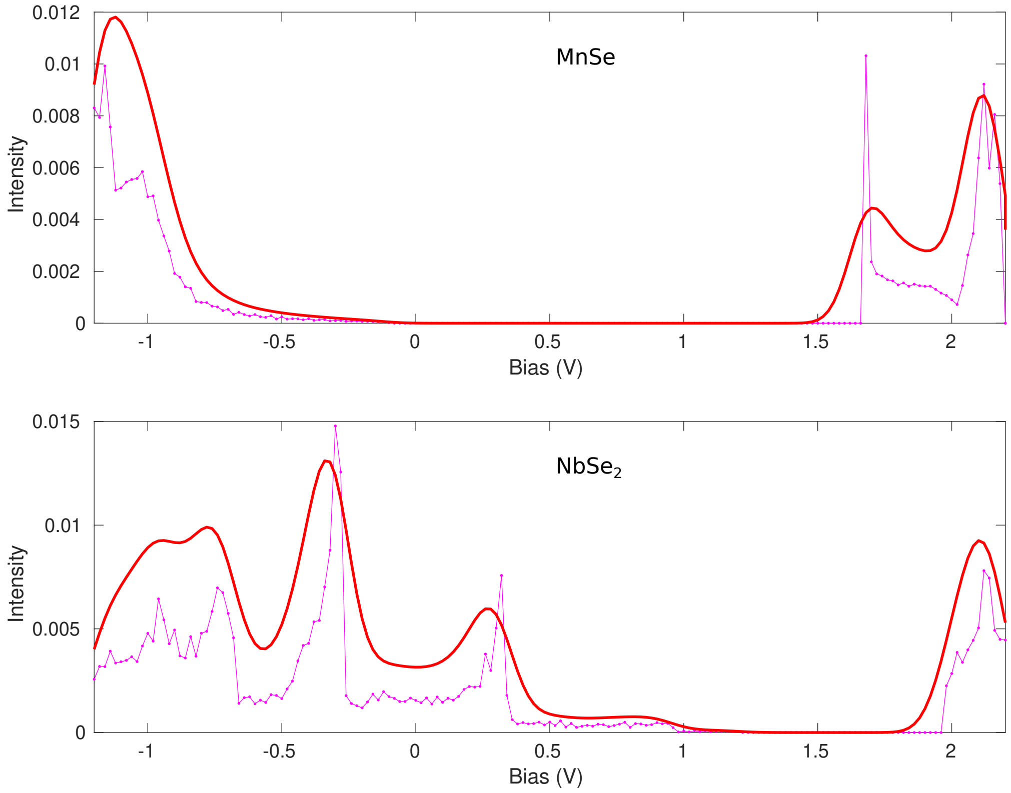

Figure S11: Simulated constant-height STS for monolayer MnSe monolayer and $NbSe_2$ trilayer, obtained by integrating the LDOS plane 2.5 Å from the surface Se atoms with a very dense k-point meshes (150×150 for MnSe and 100×100 form $NbSe_2$).

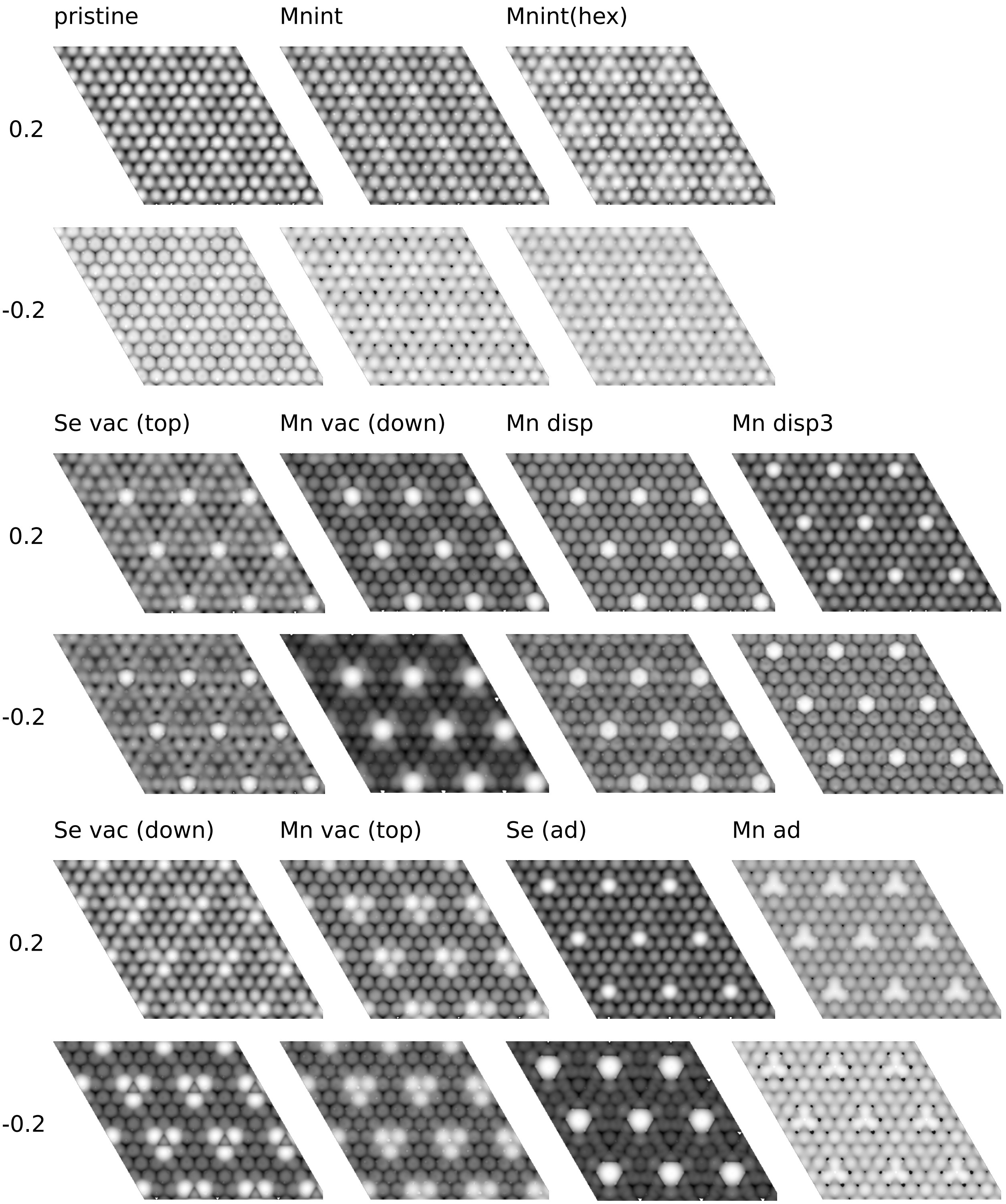

Figure S12: Simulated constant-current STM images from pristine heterostructure and from all all defective systems at small positive and negative bias.

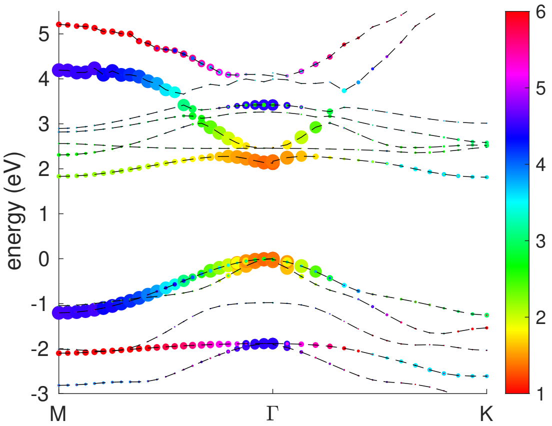

Figure S13: Band structure (dashed lines) and the oscillator strengths (transition dipole moment squared, filled circles). The size of the circle represent the oscillator strength and the color the energy difference between the initial and final states.

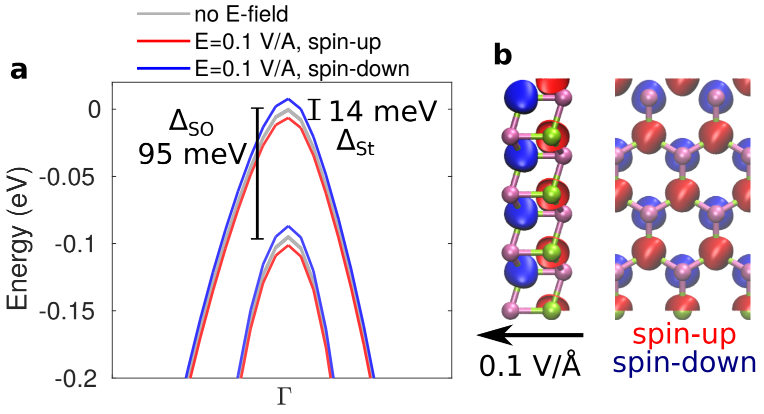

Figure S14: (a) The splitting of the monolayer Mn_2Se_2 VBM under applied 0.1 V/Å out-of-plane electric field. Spin-orbit coupling is included. (b) Spin-up and -down channels of the partial charge density of the VBM state.

Table S4: Structural parameters and energetic of Mn-Se layers considered in this work. Here, M, FE and t represents total magnetic moment per Mn atom, formation energy per atom and thickness of the layer, respectively.

Mn-Se	Lattice Parameter		Angle	Average Bond Distance			M	FE	t
Layer	a(Å)	b(Å)	γ°	Mn-Mn(Å)	Mn-Se(Å)	Se-Se(Å)	μ_B	eV/atom	Å
Mn_5Se_2	4.77	3.77	113	2.84	2.70	4.44	0.85	-1.13	5.64
Mn_4Se_2	4.20	4.18	90	3.01	2.60	4.19	4.50	-1.08	5.29
Mn_5Se_3	3.90	3.97	90	2.96	2.68	3.90	2.69	-1.15	8.45
Mn_3Se_2	3.60	3.60	90	2.99	2.59	3.60	4.20	-1.16	4.93
Mn_4Se_3	4.37	4.37	60	3.14	2.61	4.37	2.50	-1.15	6.66
Mn_2Se_2	4.28	4.28	120	3.69	2.59	4.28	0.00	-1.45	3.35
Mn_3Se_4	3.78	3.78	120	3.91	2.68	3.78	3.25	-1.20	4.93
Mn_2Se_3	3.72	3.72	120	3.81	2.63	3.72	4.00	-1.12	5.81
$MnSe_2$	3.58	2.58	120	3.58	2.52	3.58	3.00	-0.91	2.88
α-MnSe	3.87	3.87	60	3.87	2.74	3.87	0.00	-1.47	Bulk
H-MnSe	4.24	4.24	120	4.21	2.59	4.21	0.00	-1.47	Bulk

Table S5: Information about magnetic states of selected Mn-Se phases. $\Delta E = [E(\mathrm{AFM}) - E(\mathrm{FM})]/n(\mathrm{Mn})$ is the energy difference between AFM and FM states (negative value means AFM state is more stable) normalized per Mn atom n(Mn) and m is the local magnetic moment in Mn/Se atoms for FM and AFM states. For Mn_2Se_2, Mn_3Se_4, and Mn_2Se_3, AFM configuration corresponds to opposite spin orientation in the neighboring Mn layers of the unit cell. For $MnSe_2$, we adopted a lateral striped phase.

phase	ΔE	m(FM)	m(AFM)
Mn_2Se_2	-0.232	4.51/0.07	±4.38/±0.00
Mn_3Se_4	0.067	4.2/0.1	±4.2/±0.1
Mn_2Se_3	0.208	4.05/±0.07	±4.05/±0.20
$MnSe_2$	0.065	3.43/-0.21	±3.51/±0.07
